# DBT Modulates Copper‐Dependent Cuproptosis‐Related Injury in Experimental Alcoholic Cardiomyopathy

**DOI:** 10.1111/jcmm.71421

**Published:** 2026-10-30

**Authors:** Zhihao Pan, Liuming Gao, Yi Ling, Xianfeng Cen

**Affiliations:** ^1^ Department of Cardiology Zhongnan Hospital of Wuhan University Wuhan Hubei China; ^2^ Medical Research Institute of Wuhan University Wuhan Hubei China; ^3^ Department of Cardiovascular Surgery Lihuili Hospital Affiliated to Ningbo University Ningbo Zhejiang China

**Keywords:** alcohol, alcoholic cardiomyopathy, bioinformatics, cuproptosis, DBT

## Abstract

Alcoholic cardiomyopathy (ACM) is a severe cardiovascular complication of chronic alcohol consumption, but its underlying mechanisms remain incompletely understood. Cuproptosis is a recently identified copper‐dependent form of regulated cell death; however, its role in ACM remains unclear. In this study, male C57BL/6 mice were fed an ethanol‐containing Lieber‐DeCarli liquid diet to establish an ACM model, and RNA sequencing combined with bioinformatics analysis was performed to identify cuproptosis‐related genes. Among the candidate genes, dihydrolipoamide branched‐chain transacylase E2 (DBT) showed a consistent trend of downregulation across both independent RNA‐seq cohorts, and was further validated by qRT‐PCR and Western blotting. ACM mice exhibited increased serum and cardiomyocyte copper content, altered cuproptosis‐related markers and impaired cardiac function. In AC16 cells, DBT knockdown induced copper overload, cuproptosis‐related molecular alterations and mitochondrial dysfunction, which were markedly alleviated by the copper chelator tetrathiomolybdate. Conversely, DBT overexpression attenuated EtOH‐induced copper overload, cuproptosis‐related molecular alterations and mitochondrial dysfunction, with effects similar to those of tetrathiomolybdate. These findings suggest that DBT regulates ACM‐related cardiomyocyte injury through cuproptosis‐associated processes at the cellular level and may represent a potential therapeutic target for alcohol‐induced myocardial injury.

## 1 Introduction

Alcohol abuse and dependence are critical global public health concerns, ranking as the seventh leading risk factor for death and disability worldwide [1]. Long‐term excessive alcohol consumption can lead to myocardial cell injury and ultimately result in nonischemic dilated cardiomyopathy, which is referred to as alcoholic cardiomyopathy (ACM) [2]. Characterized by systolic dysfunction, ventricular dilation, apoptosis and fibrosis, ultimately progressing to heart failure [3].

Copper, an essential trace element, is involved in the mitochondrial electron transport chain (mtETC), the tricarboxylic acid (TCA) cycle and the production of reactive oxygen species (ROS) [4]. Maintaining an appropriate intracellular copper level is essential for the cardiovascular system [5]. However, excessive intracellular accumulation of copper can promote oxidative stress, impair mitochondrial function and disrupt protein homeostasis [6, 7] Recent studies have identified cuproptosis as a newly discovered regulated cell death distinct from necrosis, apoptosis, pyroptosis and ferroptosis. Cuproptosis is triggered by the direct binding of copper to lipoylated proteins within the TCA cycle, leading to proteotoxic stress through the aggregation of lipoylated proteins and the loss of iron–sulfur cluster proteins, ultimately culminating in cell death [8].

Several lines of evidence suggest that cuproptosis may be involved in the pathogenesis of ACM: (1) The heart relies on mitochondrial metabolism, which is impaired in ACM with oxidative stress and ROS accumulation [9], suggesting cuproptosis may contribute by disrupting mitochondrial function [8]. (2) Serum copper levels are significantly elevated in patients with ACM [10, 11], which aligns with the core mechanism of cuproptosis‐copper overload. (3) Cuproptosis has been implicated in multiple cardiovascular diseases, including myocardial infarction, heart failure, dilated cardiomyopathy, diabetic cardiomyopathy and myocardial ischemia–reperfusion injury [12, 13, 14, 15, 16, 17]. (4) Copper chelators can rescue cells from cuproptosis. Trientine, a copper chelator, has demonstrated therapeutic potential in diabetes‐induced myocardial injury [18]. Based on the above evidence, we hypothesize that cuproptosis may represent a promising novel therapeutic mechanism and potential target for the treatment of ACM.

## 2 Materials and Methods

### 2.1 Animals and ACM Model

The animal experimental protocol was approved by the Experimental Animal Center of Medical Research Institute, Wuhan University. ACM mice model was established by feeding with an ethanol‐containing Lieber‐DeCarli liquid diet [19, 20, 21]. Previous studies have shown that mice with lower body weights exhibit significantly higher mortality rates [19]. Thirty male C57BL/6 mice (6–8 weeks, approximately 25 g; Shubeili Biotechnology, China) were randomly assigned to ethanol‐fed (*n* = 18) and control (*n* = 12) groups. More mice were allocated to ethanol feeding to account for anticipated mortality. Mice received an ethanol‐containing Lieber‐DeCarli liquid diet (710,260, Dyets, USA) or an isocaloric ethanol‐free diet (710027, Dyets, USA). Mice in the alcohol group first underwent a 5‐day adaptive feeding period, during which the ethanol concentration (%, vol/vol) was gradually increased from 1% to 5% [19]. This was followed by a standardized feeding protocol in which the ethanol concentration (%, w/v) was increased every two weeks: 4.8% during weeks 1–2, 5.0% during weeks 3–4, 5.2% during weeks 5–6 and maintained at 5.4% during weeks 7–10 [20]. Three mice were housed per cage, and liquid diets were freshly prepared every 3 days. A fresh diet (50 mL per cage, 50 kcal, 1 kcal/mL) was administered daily. Each mouse consumed approximately 15 mL of the diet per day [22]. The same feeding protocol was followed for the control group. Since the control diet did not contain ethanol, an equivalent amount of energy was supplemented with maltodextrin (M305460, Aladdin, China) to ensure caloric balance. The adaptive feeding period was used to improve tolerance to the liquid diet and ethanol, thereby reducing early mortality and improving model stability.

### 2.2 Blood Metabolite Analysis

Serum samples of mice were collected after the experiment. Blood alcohol concentration (BAC) was measured using an ethanol assay kit (BC6035, Solarbio, China). Serum alanine aminotransferase (ALT) and aspartate aminotransferase (AST) levels were determined using commercial kits (C009‐2‐1 and C010‐2‐1, Nanjing Jiancheng, China). Serum B‐type natriuretic peptide (BNP) levels were measured using an ELISA kit (CSB‐E07971m, Cusabio Biotech Co. Ltd., China) and serum copper content was quantified using a copper content detection kit (E‐BC‐K300‐M, Elabscience, China).

### 2.3 Echocardiographic Assessment of Cardiac Function

After 10 weeks of feeding, cardiac structure and function were assessed using a high‐resolution small‐animal ultrasound imaging system (Vevo 2100, Fujifilm VisualSonics, Canada) [23, 24]. To minimize the effects of anaesthesia on myocardial contractile function and heart rate, parameters related to left ventricular systolic function were mainly measured in conscious mice. Parasternal short‐axis M‐mode images were acquired, and left ventricular ejection fraction (LVEF), left ventricular fractional shortening (LVFS), left ventricular internal diameter at end‐systole (LVIDs) and left ventricular internal diameter at end‐diastole (LVIDd) were measured at the papillary muscle level.

Because Doppler‐derived indices of diastolic function are sensitive to body movement and respiratory artefacts in mice, diastolic function was assessed under light inhalational anaesthesia with isoflurane. During image acquisition, mice were maintained under 1.5% isoflurane anaesthesia and placed in the supine position on a temperature‐controlled imaging platform to maintain body temperature, with heart rate monitored throughout the examination. Left ventricular diastolic function was evaluated from the apical four‐chamber view. Pulsed‐wave Doppler was used to measure the peak early transmitral inflow velocity (E wave), and tissue Doppler imaging was used to measure the early diastolic mitral annular velocity (*e′*) and late diastolic mitral annular velocity (*a′*). The *E*/*e′* ratio was then calculated. All echocardiographic parameters were averaged from at least three consecutive cardiac cycles.

### 2.4 Haematoxylin and Eosin Staining and Masson's Trichrome Staining

Heart tissues were fixed in 4% paraformaldehyde solution for at least 24 h, followed by dehydration and paraffin embedding. Four‐chamber cross‐sections were obtained. Haematoxylin and eosin (H&E) staining and Masson's trichrome staining were performed to evaluate the myocardial structural changes and fibrosis.

### 2.5 Transcriptome Sequencing Analysis

Total RNA was extracted from the left ventricular tissue of control and ACM model mice using an RNA extraction kit (TSP413, Tsingke, China), and RNA concentration was quantified using a NanoDrop spectrophotometer. RNA integrity was assessed using the Agilent 2100 Bioanalyzer, and only samples with an RNA integrity number (RIN) ≥ 7.0 were selected. High‐quality RNA samples were used to construct cDNA libraries, which were then sequenced on the Illumina NovaSeq 6000 platform. Clean reads were mapped to the mouse reference genome (GRCm38/mm10) using HISAT2. Gene‐level read counts were quantified using featureCounts, generating a raw count matrix for downstream differential expression analysis. The overall distribution of log10(FPKM) values across RNA‐seq samples was comparable among groups, indicating consistent sequencing quality and expression distribution (Figure S1).

To improve the robustness of transcriptomic screening, an independent RNA‐seq validation cohort (Figure S2) was additionally generated and processed using the same sequencing and bioinformatics pipeline.

### 2.6 Bioinformatics Analysis

Given the relatively small RNA‐seq sample sizes, *p* values from differential‐expression analyses were adjusted using the Benjamini‐Hochberg method to control for multiple testing, with FDR < 0.05 considered statistically significant. Because the transcriptomic analysis was primarily exploratory, |logFC| > 0.2 and *p* < 0.05 were retained as screening criteria for potential cuproptosis‐related candidates [13, 25, 26]. Genes meeting the exploratory criteria but not FDR < 0.05 were defined as exploratory candidates rather than FDR‐significant differentially expressed genes (FDR‐significant DEGs). Differential‐expression analysis and FDR correction were performed separately for the discovery and independent validation cohorts.

We obtained 86 cuproptosis‐related genes (CRGs) from published literature [14, 27, 28, 29](Table S1). The intersection between the identified exploratory candidates and CRGs was analysed to obtain cuproptosis‐related exploratory candidates and a Venn diagram was generated. Volcano plots and heatmaps were generated using ggplot2. Functional enrichment analyses, including Gene Ontology (GO) and Kyoto Encyclopedia of Genes and Genomes (KEGG) pathway analyses, were performed for the cuproptosis‐related exploratory candidates using clusterProfiler and org.Mm.eg.db. Significant pathways with *p*‐value < 0.05 were visualized. The protein–protein interaction (PPI) network was constructed using the STRING database with a minimum interaction score of 0.15 [30]. The network was visualized using Cytoscape [31]. Additionally, violin plots were generated using ggpubr to visualize the expression of intersection genes between the control group and the alcohol group. GSEA and GSVA were performed using R to explore pathways associated with DBT expression.

### 2.7 Adult Mouse Cardiomyocyte Isolation

Adult mouse cardiomyocytes were isolated as previously described [32, 33]. Briefly, mice were anaesthetised, and the hearts were rapidly excised. After aortic cannulation, the hearts were subjected to retrograde perfusion with calcium‐free perfusion buffer followed by digestion buffer containing Type II collagenase. After sufficient enzymatic digestion, the left ventricular tissue was dissected and gently triturated to release single cardiomyocytes. The cell suspension was filtered and allowed to settle by low‐speed sedimentation, followed by gradual calcium reintroduction. Rod‐shaped adult mouse cardiomyocytes with clear striations and intact morphology were used for subsequent experiments (Figure S3).

### 2.8 AC16 Cell Culture and Transfection

Based on previous studies, AC16 cells were used to establish the in vitro cardiomyocyte injury model [34, 35, 36]. The AC16 cell line was obtained from Procell (CL‐0790, China). Cells were cultured in DMEM supplemented with 10% fetal bovine serum (FBS) and 1% penicillin–streptomycin under standard conditions (37°C, 5% CO_2_).

When the cells reached approximately 70%–80% confluence, DBT knockdown was performed using DBT siRNA (si‐DBT) and negative control siRNA (si‐NC), whereas DBT overexpression was achieved using the pcDNA3.1(+)‐DBT plasmid and empty pcDNA3.1(+) vector. Transfection was performed according to the manufacturer's instructions, and the overexpression plasmids were transfected using Lipofectamine 3000 (BL632B, Biosharp, China). After transfection, cells were cultured for an additional 24–48 h before subsequent pharmacological treatment or sample collection.

### 2.9 Cell Treatment and Experimental Grouping

After 24 h of transfection, pharmacological interventions were applied for an additional 24 h.

To evaluate the effects of DBT knockdown on cardiomyocyte injury and cuproptosis‐related phenotypes, AC16 cells were divided into the Control, si‐DBT, si‐DBT+TTM and si‐DBT+MitoQ groups. Tetrathiomolybdate (TTM; 323,446, Sigma‐Aldrich, USA), a specific copper chelator, was used at 20 μM to reduce copper burden and inhibit cuproptosis [8, 37]. Mitoquinone mesylate (MitoQ; M426202, Aladdin, China), a mitochondria‐targeted antioxidant, was used at 1 μM to attenuate mitochondrial oxidative stress [38, 39].

To establish an in vitro alcoholic cardiomyopathy cell model and further evaluate the protective role of DBT, AC16 cells were exposed to 500 mM ethanol for 24 h [40, 41]. The experimental groups included Control, EtOH, EtOH+oe‐DBT, EtOH+TTM, EtOH+Z‐VAD, EtOH+Fer‐1 and EtOH+3‐MA. TTM, Z‐VAD‐FMK (Z‐VAD; C1202, Beyotime, China) [37, 42], Ferrostatin‐1 (Fer‐1; F129882, Aladdin, China) [37, 42] and 3‐Methyladenine (3‐MA; M129496, Aladdin, China) [43, 44] were used to interfere with cuproptosis, apoptosis, ferroptosis and autophagy‐related processes, respectively. The drug concentrations were as follows: TTM, 20 μM; Z‐VAD, 20 μM; Fer‐1, 10 μM; and 3‐MA, 5 mM.

### 2.10 CCK‐8 Cell Proliferation Assay

AC16 cells were seeded in 96‐well plates and cultured until they reached approximately 80% confluence. Cell viability was evaluated using the Cell Counting Kit‐8 (CCK‐8) assay (C0037, Beyotime, China). Briefly, 10 μL of CCK‐8 solution was added to each well and incubated for 2 h, and absorbance was measured at 450 nm using a microplate reader.

### 2.11 Intracellular Copper Content Assay

Intracellular copper content was measured using a Copper Content Detection Kit (E‐BC‐K300‐M, Elabscience, China). Briefly, isolated cardiomyocytes from ACM mice and treated AC16 cells were lysed and prepared according to the manufacturer's instructions. Copper content was measured and normalized to the total protein concentration. Intracellular copper content was expressed as μmol/g protein.

### 2.12 Mitochondrial Functional Assays

Intracellular ROS levels, mitochondrial membrane potential, ATP content and the NAD^+^/NADH ratio were measured using a ROS Assay Kit (S0033S, Beyotime, China), JC‐1 Mitochondrial Membrane Potential Assay Kit (C2006, Beyotime, China), ATP Assay Kit (S0026, Beyotime, China) and NAD^+^/NADH Assay Kit (S0175, Beyotime, China), respectively, according to the manufacturer's instructions.

### 2.13 Immunofluorescence Assay

Immunofluorescence analysis was performed to detect DLAT oligomerization in cardiac tissues or AC16 cells. Samples were fixed and incubated with a primary antibody against DLAT (13426–1‐AP, Proteintech, China), followed by a fluorescently labelled secondary antibody (A0423, Beyotime, China). Nuclei were counterstained with DAPI (G1407, Servicebio, China). Fluorescence images were captured using a Leica Thunder Imager microscope (Leica Microsystems, Germany).

### 2.14 qRT‐PCR

Total RNA was extracted from mouse heart tissues or AC16 cells using the RNA extraction kit (TSP413, Tsingke, China). cDNA was synthesized using a cDNA synthesis kit (G3337, Servicebio, China), and qRT–PCR was performed using the BeyoFast SYBR Green qPCR Mix (CW0957M, Cwbio, China). The mRNA levels of FDX1, SLC31A1, HSP70, DBT, CP and PDK1 were quantified using the 2^−ΔΔCt^ method, with GAPDH as the internal reference gene. Primer sequences are listed in Table S2.

### 2.15 Western Blot Analysis

The primary antibodies used in this study included FDX1, HSP70, SLC31A1, DLAT, DBT and GAPDH. Detailed information on the primary antibodies, such as manufacturers and dilutions, is provided in Table S3. Protein band intensities were quantified using ImageJ (National Institutes of Health, USA) in a blinded manner.

### 2.16 Statistical Analysis

Data are expressed as mean ± SD unless otherwise indicated and analysed using GraphPad Prism version 9.5.0 (GraphPad Software, San Diego, CA, USA). Statistical significance between two groups was determined by a two‐tailed unpaired *t*‐test. For multiple groups with equal variances, statistical significance was evaluated using one‐way ANOVA. *p* < 0.05 was considered statistically significant. The *n* value represents the number of animals or independent biological replicates, as indicated in the corresponding figure legends. Animals and cells were randomly assigned to experimental groups. Quantitative analyses of histology, immunofluorescence, Western blotting and echocardiographic parameters were performed in a blinded manner after sample or image coding. No formal a priori power calculation was performed; sample sizes were determined based on previous studies, preliminary experience and experimental feasibility, and are indicated in the corresponding figure legends.

## 3 Results

### 3.1 Establishment of the ACM Mouse Model

During the 10‐week modelling period, the ethanol concentration was gradually increased every 2 weeks (Figure 1A). Three mice were housed per cage (Figure 1B). Body weight was recorded weekly. Although body weight increased in both groups after the initiation of feeding, the ethanol group presented slower weight gain and significantly lower body weight starting from week six compared to the control group (Figure 1C). At 10 weeks post‐modelling, serum ALT, AST, ethanol and BNP levels were measured; these parameters were significantly elevated (Figure 1D) in the ethanol group, indicating successful alcohol exposure and cardiac injury.

**FIGURE 1:**
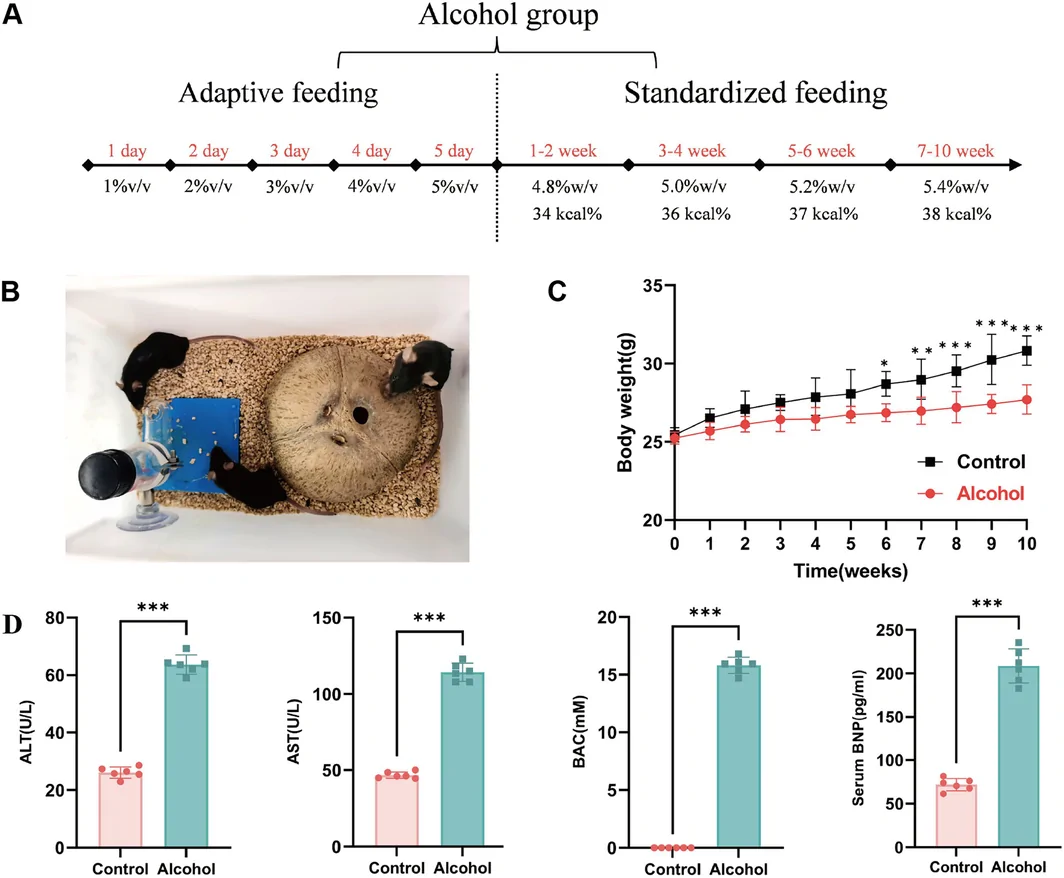
Ethanol‐containing Lieber‐DeCarli liquid diet feeding. (A) Schematic diagram of the mouse feeding protocol (showing the gradient change in alcohol concentration). (B) Representative cage and feeding‐tube setup. (C) Weekly monitoring of mouse body weight. (D) Serum levels of ALT, AST, BAC and BNP in mice (*n* = 6) (Data are presented as mean ± SD, *p < 0.05, **p < 0.01, ***p < 0.001).

### 3.2 Cardiac Injury in the Alcohol Group

After 10 weeks of modelling, transthoracic echocardiography was performed. Compared with the control group, mice in the ethanol‐treated group exhibited cardiac chamber dilation and impaired contractile function (Figure 2A,B), with significantly reduced LVEF and LVFS, along with markedly increased LVID in both diastole and systole (Figure 2C). Left ventricular diastolic function was further assessed using mitral inflow Doppler and tissue Doppler imaging. Compared with the control group, ACM mice showed no significant difference in heart rate but exhibited a markedly reduced early diastolic mitral annular velocity (*e′*) and an increased *E*/*e′* ratio (Figure 2D,E), indicating impaired left ventricular diastolic function. Morphometric analysis showed decreased body weight, increased heart weight and an elevated liver‐to‐body weight ratio in the alcohol group (Table 1). Histologic analysis revealed disorganized myocardial architecture, left ventricular chamber enlargement, wall thinning and inflammatory cell infiltration in the alcohol group. Masson's trichrome staining further revealed extensive collagen deposition in the myocardial interstitium (Figure 2F), indicating the presence of myocardial fibrosis. Collectively, these findings confirm the successful establishment of the alcoholic cardiomyopathy (ACM) model.

**FIGURE 2:**
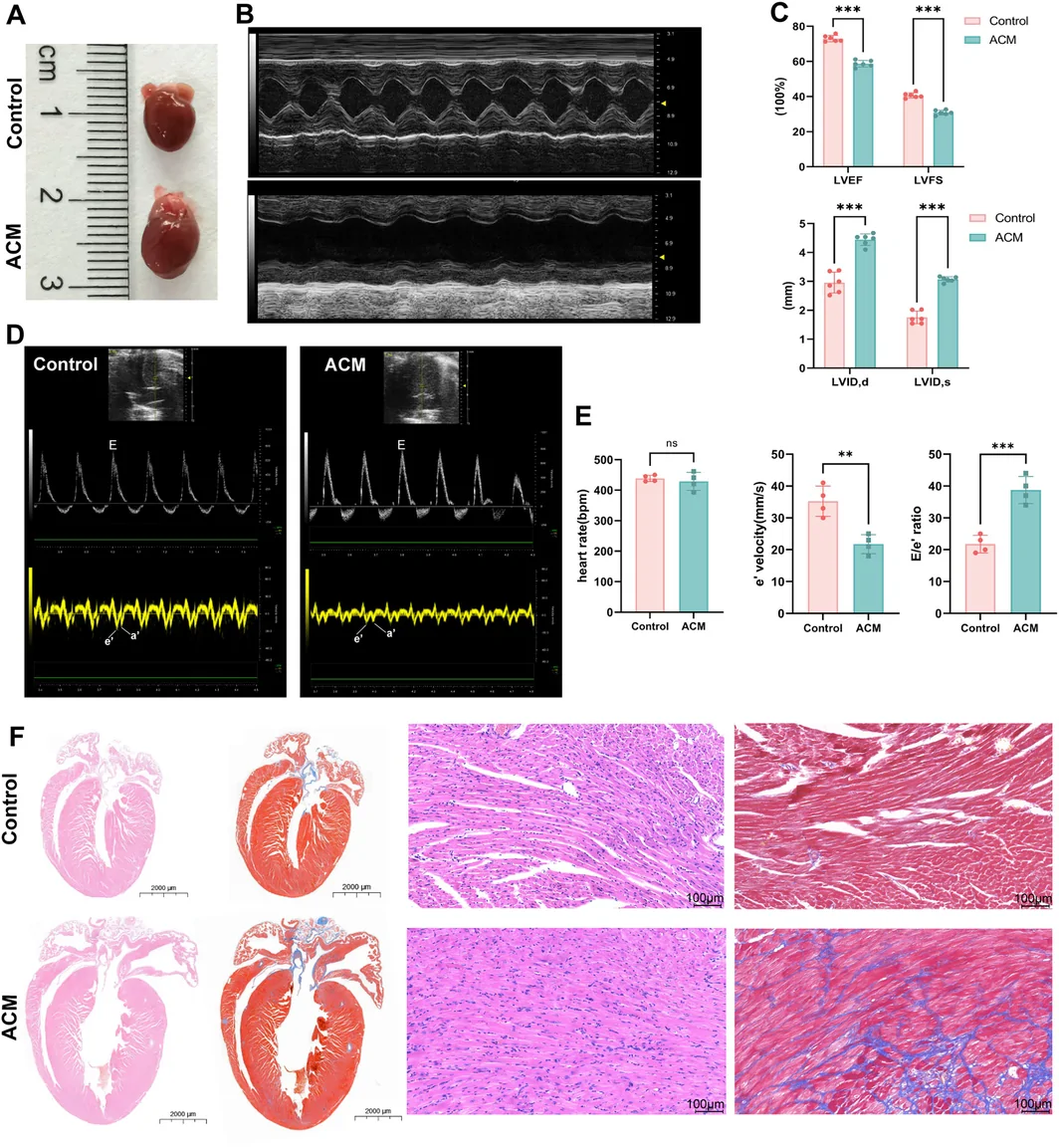
Cardiac injury in ACM mice. (A) Representative Cardiac Tissue Samples from Two Groups. (B) Echocardiographic Analysis (*n* = 3). (C) In the ACM group, both LVEF and LVFS were significantly decreased, while LVID in diastole and systole was significantly increased (*n* = 6). (D) Representative mitral inflow Doppler and tissue Doppler images. (E) Quantification of heart rate, *e′* velocity and *E*/*e′* ratio (*n* = 4). (F) HE and Masson staining revealed disorganized myocardial cell arrangement, enlarged cardiac chambers, and reduced wall thickness in ACM mice. Masson staining also showed a marked increase in interstitial collagen fibres. Scale bars: 2000 μm and 100 μm (*n* = 3) (Data are presented as mean ± SD. ns, not significant; *p < 0.05, **p < 0.01, ***p < 0.001).

**TABLE 1:** General features of C57 mice with or without chronic alcohol treatment.

Parameter	Control (*n* = 10)	Alcohol (*n* = 10)
Body weight (g)	31.20 ± 0.83	27.48 ± 0.73***
Tibia length (cm)	1.92 ± 0.058	1.94 ± 0.074 ns
Heart weight (mg)	147.27 ± 2.56	170.52 ± 3.55***
Liver weight (mg)	1276.5 ± 26.87	1317.5 ± 42.85 ns
HW/BW (mg/g)	4.73 ± 0.16	6.22 ± 0.24***
LW/BW (mg/g)	41.02 ± 1.80	47.99 ± 1.65***
HW/TL (mg/cm)	76.77 ± 1.87	88.09 ± 4.19**
LW/TL (mg/cm)	665.86 ± 23.88	680.33 ± 33.67 ns

*Note:* Mean ± SEM.

Abbreviations: BW, body weight; HW, heart weight; LW, liver weight; TL, Tibia length; ns, not significant.

** *p* < 0.01.

*** *p* < 0.001.

### 3.3 Transcriptomic Bioinformatics Analysis

Left ventricular tissues from four Control and four ACM mice were subjected to RNA‐seq analysis. Using the predefined screening criteria (|logFC| > 0.2 and *p* < 0.05), 2485 exploratory candidates were identified and visualized by heatmap (Figure 3A). Benjamini‐Hochberg correction was further applied, with FDR < 0.05 considered statistically significant after multiple testing correction. Intersection of the 2485 exploratory candidates with 86 known CRGs identified seven cuproptosis‐related exploratory candidates: VEGFA, CP, SQSTM1, SLC31A2, DBT, PDK1 and SERPINE1. The volcano plot, PPI network and Venn diagram are shown in Figure 3B–D. Among these candidates, VEGFA and CP remained significant after FDR correction (FDR = 0.024890 and 0.024924), whereas the remaining genes did not reach FDR < 0.05 and were therefore considered exploratory, hypothesis‐generating candidates rather than FDR‐significant DEGs (Table S4).

**FIGURE 3:**
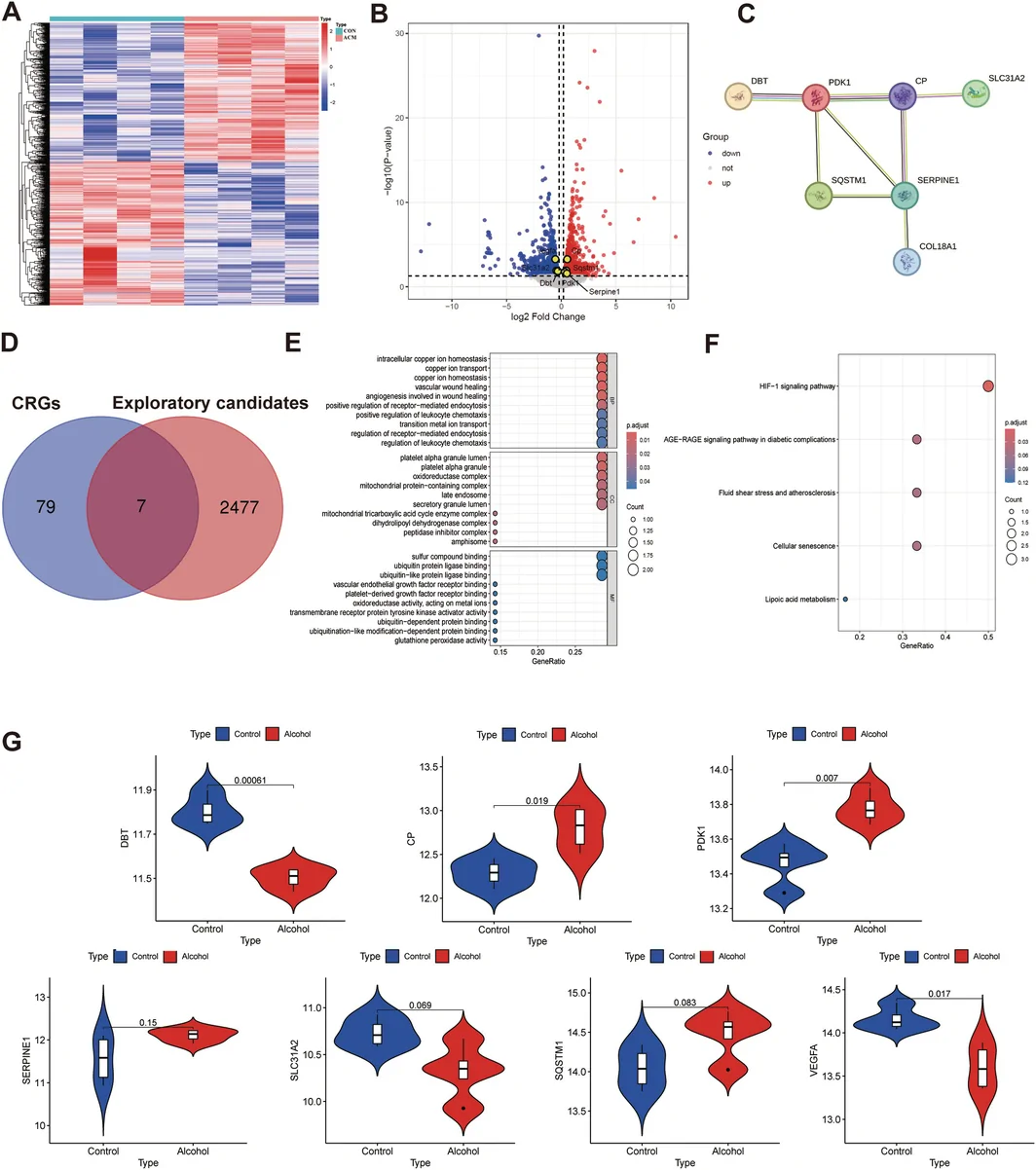
Bioinformatics analysis of cuproptosis‐related genes in ACM. (A) Heatmap displaying the exploratory candidates between the two groups (*n* = 4 per group). (B) Volcano plot of transcriptome sequencing analysis showing exploratory candidates between the two groups (cuproptosis‐related exploratory candidates highlighted). (C) Protein–protein interaction (PPI) network of the 7 cuproptosis‐related exploratory candidates. (D) Venn diagram showing the overlap between exploratory candidates and CRGs. (E) GO enrichment analysis of the 7 cuproptosis‐related exploratory candidates (BP: Biological Process, CC: Cellular Components, MF: Molecular Function). (F) KEGG pathway enrichment analysis of the 7 cuproptosis‐related exploratory candidates. (G) Violin plots of expression levels of the cuproptosis‐related exploratory candidates (*p* values are indicated in the panels).

GO and KEGG enrichment analyses were performed on these seven genes (Figure 3E,F). GO terms were mainly enriched in copper ion homeostasis and transport, mitochondrial protein‐containing complexes, oxidoreductase complexes and sulfur compound binding, while KEGG analysis highlighted the HIF‐1 signalling pathway and cellular senescence. Violin plots showed nominal *p* < 0.05 for DBT, CP and PDK1 (Figure 3G). Therefore, these three genes were selected for subsequent qRT‐PCR and WB validation.

### 3.4 DBT Gene

qRT‐PCR and Western blot analyses showed that, among the three candidate genes, only DBT exhibited a significant difference between the control and ACM groups at both the mRNA and protein levels (Figure 4A,B). DBT was consistently downregulated in both the discovery cohort (logFC = −0.32154, *p* = 0.013933, FDR = 0.198855) and validation cohort (logFC = −0.32434, *p* = 0.006849, FDR = 0.111048) (Figure S2; Table S4), but did not reach FDR significance in either cohort. Thus, DBT was considered an exploratory, hypothesis‐generating candidate with reproducible downregulation rather than an FDR‐significant DEG. Gene set enrichment analysis (GSEA) further indicated that DBT is associated with several cardiomyopathy‐related pathways, including arrhythmogenic right ventricular cardiomyopathy, dilated cardiomyopathy, hypertrophic cardiomyopathy and Viral Myocarditis (Figure 4C). Gene set variation analysis (GSVA) suggested a potential association between DBT and branched‐chain amino acids (BCAAs) catabolic pathways (Figure 4D). DBT encodes dihydrolipoamide branched‐chain transacylase E2, which participates in BCAA catabolism [45]. These findings motivated functional studies of DBT in cuproptosis‐related injury [8].

**FIGURE 4:**
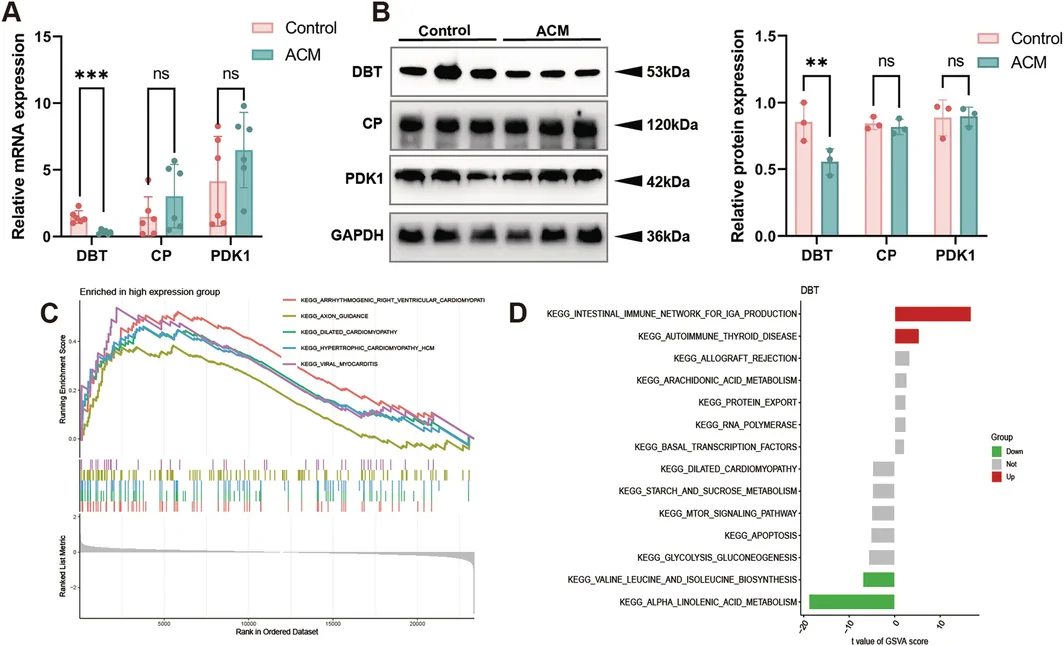
Validation and functional analysis of DBT in ACM. (A) qRT‐PCR detection of DBT, CP and PDK1 expression in mouse heart tissue (*n* = 6). (B) Western blot analysis and quantification of DBT, CP and PDK1 protein expression (*n* = 3). (C) GSEA of the DBT gene. (D) GSVA of the DBT gene (Data are presented as mean ± SD. ns, not significant; **p < 0.01, ***p < 0.001).

### 3.5 Cuproptosis‐Related Alterations in ACM

During cuproptosis, there is a loss of Fe‐S cluster proteins (in an FDX1‐dependent manner), oligomerization of lipoylated DLAT, upregulation of HSP70 [8, 37] and downregulation of SLC31A1 [17]. SLC31A1 is a key copper transporter protein that mediates copper uptake across the plasma membrane [46]. The observed downregulation of SLC31A1 during cuproptosis may represent a negative feedback response triggered by chronic copper overload [17]. Serum copper levels were significantly elevated in ACM mice (Figure 5A). To further determine whether copper burden was increased in cardiomyocytes, cardiomyocytes were isolated from the hearts of Control and ACM mice (Figure S3), and intracellular copper content was measured. The results showed that intracellular copper content was significantly increased in cardiomyocytes from the ACM group (Figure 5B), indicating copper accumulation in ACM cardiomyocytes. qRT‐PCR and Western blotting showed decreased FDX1 and SLC31A1 and increased HSP70 expression in ACM myocardium (Figure 5C,D). Western blotting and immunofluorescence also revealed increased DLAT oligomerization and aggregation, respectively (Figure 5E,F). Collectively, these findings indicate copper accumulation and cuproptosis‐related molecular alterations in ACM cardiomyocytes, suggesting that cuproptosis may be involved in ACM pathogenesis.

**FIGURE 5:**
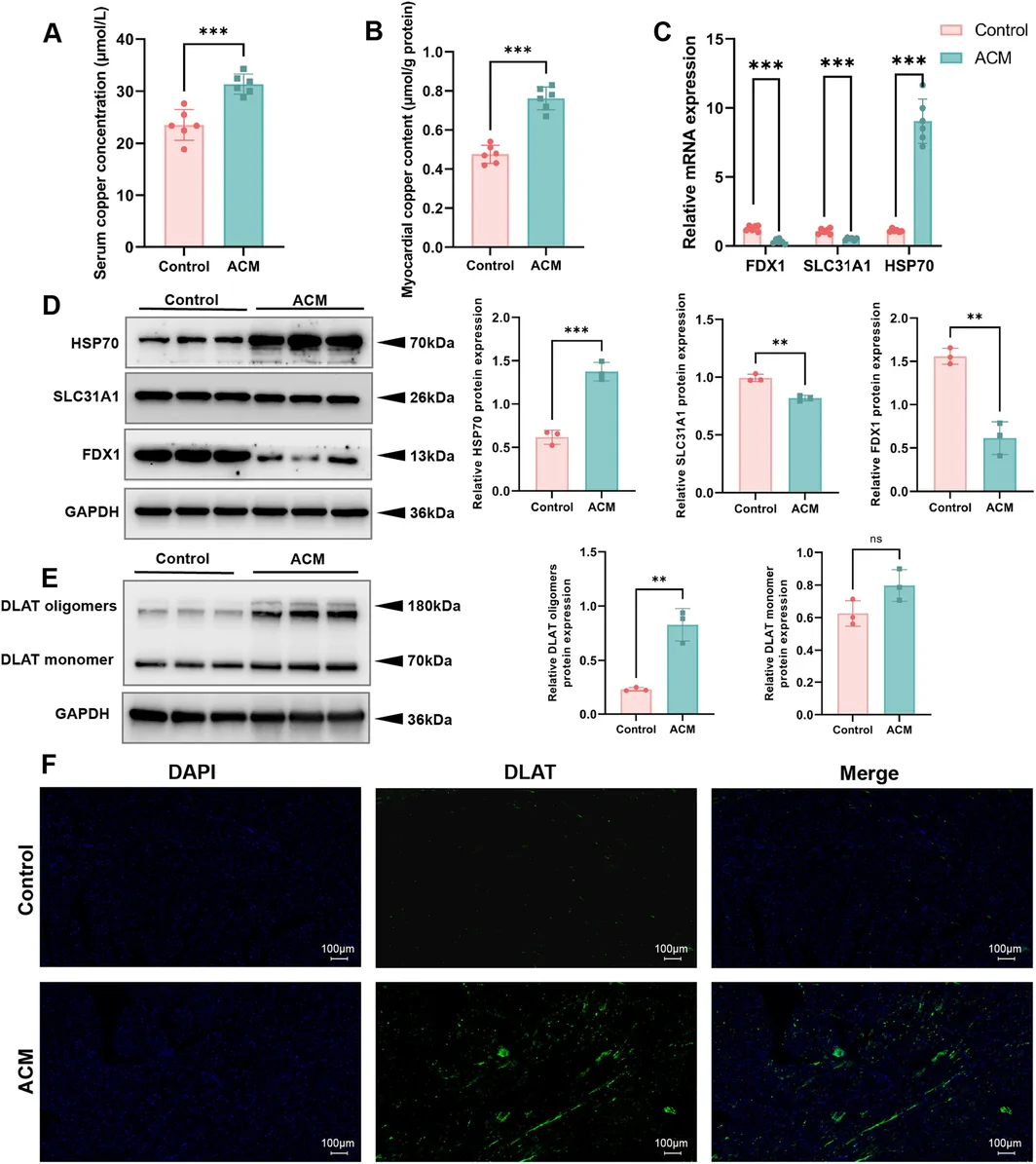
Copper accumulation and cuproptosis‐related alterations in ACM cardiomyocytes. (A) Serum copper ion concentration in mice (*n* = 6). (B) Intracellular copper content in isolated cardiomyocytes (*n* = 6). (C) qRT‐PCR detection of FDX1, SLC31A1 and HSP70 expression in mouse heart tissue (*n* = 6). (D) Western blot analysis and quantification of HSP70, SLC31A1 and FDX1 protein expression in myocardial tissue (*n* = 3). (E) Western blot analysis and quantification of DLAT oligomers and DLAT monomer in myocardial tissue (*n* = 3). (F) Representative DLAT immunofluorescence staining in cardiac tissues. Scale bar: 100 μm. (*n* = 3) (Data are presented as mean ± SD. ns, not significant; **p < 0.01, ***p < 0.001).

### 3.6 DBT Knockdown Induces Cuproptosis‐Related Injury and Mitochondrial Dysfunction in AC16 Cells

To further determine whether DBT downregulation directly contributes to cuproptosis‐related cardiomyocyte injury, DBT was knocked down in AC16 cells (Figure 6C), followed by treatment with the copper chelator TTM or the mitochondria‐targeted antioxidant MitoQ. Compared with the Control group, si‐DBT treatment significantly reduced cell viability and induced morphological damage (Figure 6A). qRT‐PCR (Figure 6B), WB (Figure 6D,E; WB quantification is shown in Figure S4B), and immunofluorescence analyses (Figure 6K) showed cuproptosis‐related alterations, accompanied by increased intracellular copper content (Figure 6G). Further analyses showed that DBT knockdown impaired mitochondrial function, as evidenced by increased ROS levels (Figure 6F), decreased intracellular ATP levels (Figure 6H), decreased NAD^+^ levels (Figure S4A), increased NADH levels (Figure S4A), a reduced NAD^+^/NADH ratio (Figure 6I), and loss of mitochondrial membrane potential (Figure 6J). TTM markedly alleviated these si‐DBT‐induced injuries. In contrast, MitoQ partially restored mitochondrial membrane potential (Figure 6J) but did not significantly improve other DBT knockdown‐induced cuproptosis‐related alterations or mitochondrial dysfunction. These results indicate that DBT downregulation induces cuproptosis‐related molecular alterations, copper overload, and mitochondrial dysfunction in AC16 cells, while the rescue effect of TTM further supports the copper‐dependent nature of DBT knockdown‐induced cardiomyocyte injury.

**FIGURE 6:**
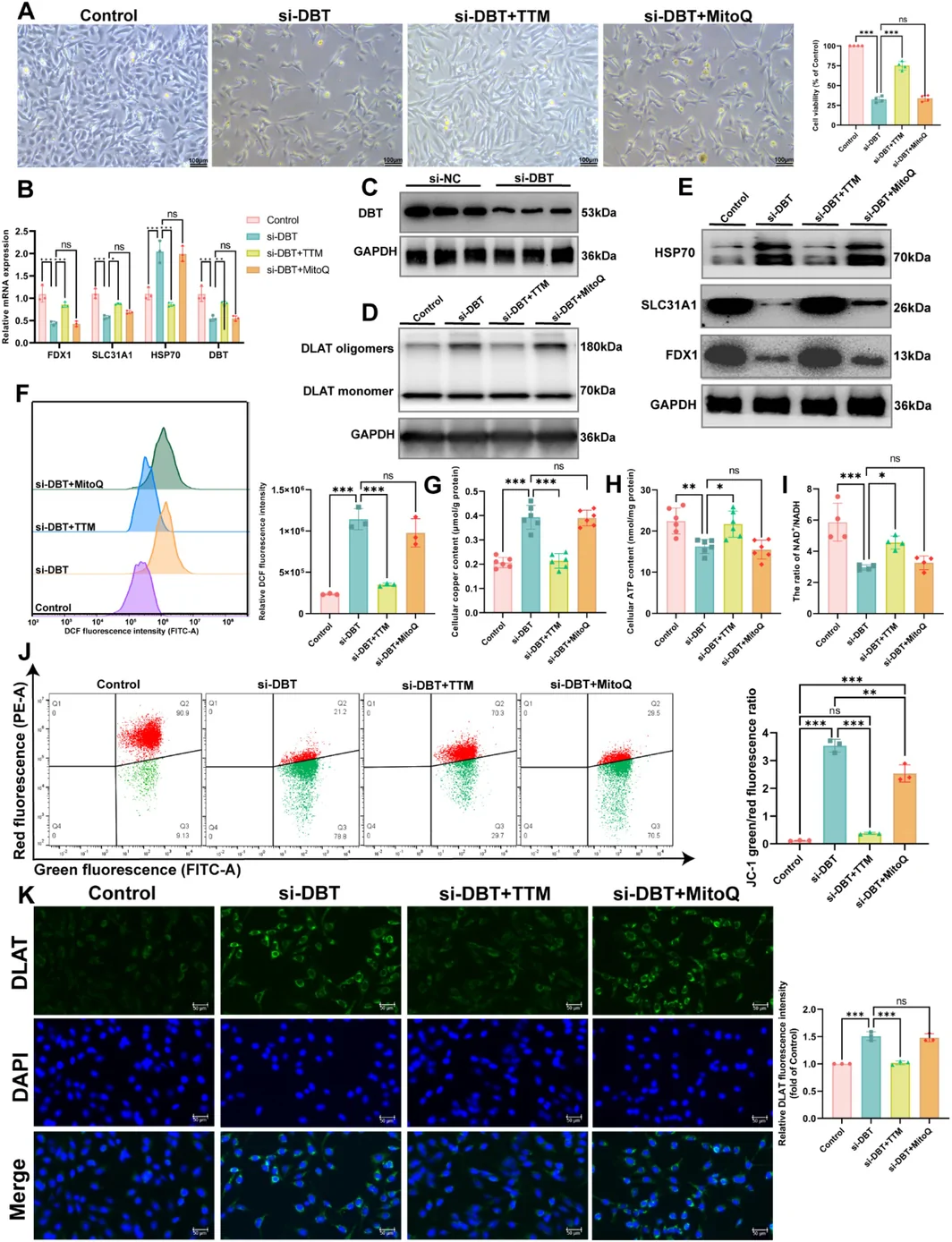
DBT knockdown induces cuproptosis‐related injury and mitochondrial dysfunction in AC16 cells. (A) Representative cell morphology and CCK‐8 analysis of cell viability in Control, si‐DBT, si‐DBT+TTM and si‐DBT+MitoQ groups. Scale bar: 100 μm (*n* = 4). (B) qRT‐PCR analysis of FDX1, SLC31A1, HSP70 and DBT mRNA expression in AC16 cells (*n* = 3). (C) Western blot validation of DBT knockdown efficiency in AC16 cells (*n* = 3). (D) Western blot analysis of DLAT oligomers and DLAT monomer (*n* = 3). (E) Western blot analysis of HSP70, SLC31A1 and FDX1 protein expression (*n* = 3, corresponding WB quantification is shown in Figure S4B). (F) Flow cytometric analysis and quantification of intracellular ROS levels (*n* = 3). (G) Intracellular copper content in AC16 cells (*n* = 6). (H) Cellular ATP content (*n* = 6). (I) Cellular NAD^+^/NADH ratio (*n* = 4). (J) JC‐1 flow cytometric analysis and quantification of mitochondrial membrane potential, expressed as the green/red fluorescence ratio (*n* = 3). (K) Representative DLAT immunofluorescence staining and fluorescence intensity quantification in AC16 cells. Scale bar: 50 μm (*n* = 3) (Data are presented as mean ± SD. ns, not significant; *p < 0.05, **p < 0.01, ***p < 0.001).

### 3.7 Alcoholic Cardiomyopathy Model in AC16 Cells

EtOH significantly reduced AC16 cell viability and caused morphological damage (Figure 7A), increased intracellular copper (Figure 7E), and induced cuproptosis‐related molecular alterations resembling those observed in ACM mice (Figure 7C,I; Figure S4D). Mitochondrial dysfunction was also observed (Figure 7D,F–H; Figure S4C).

**FIGURE 7:**
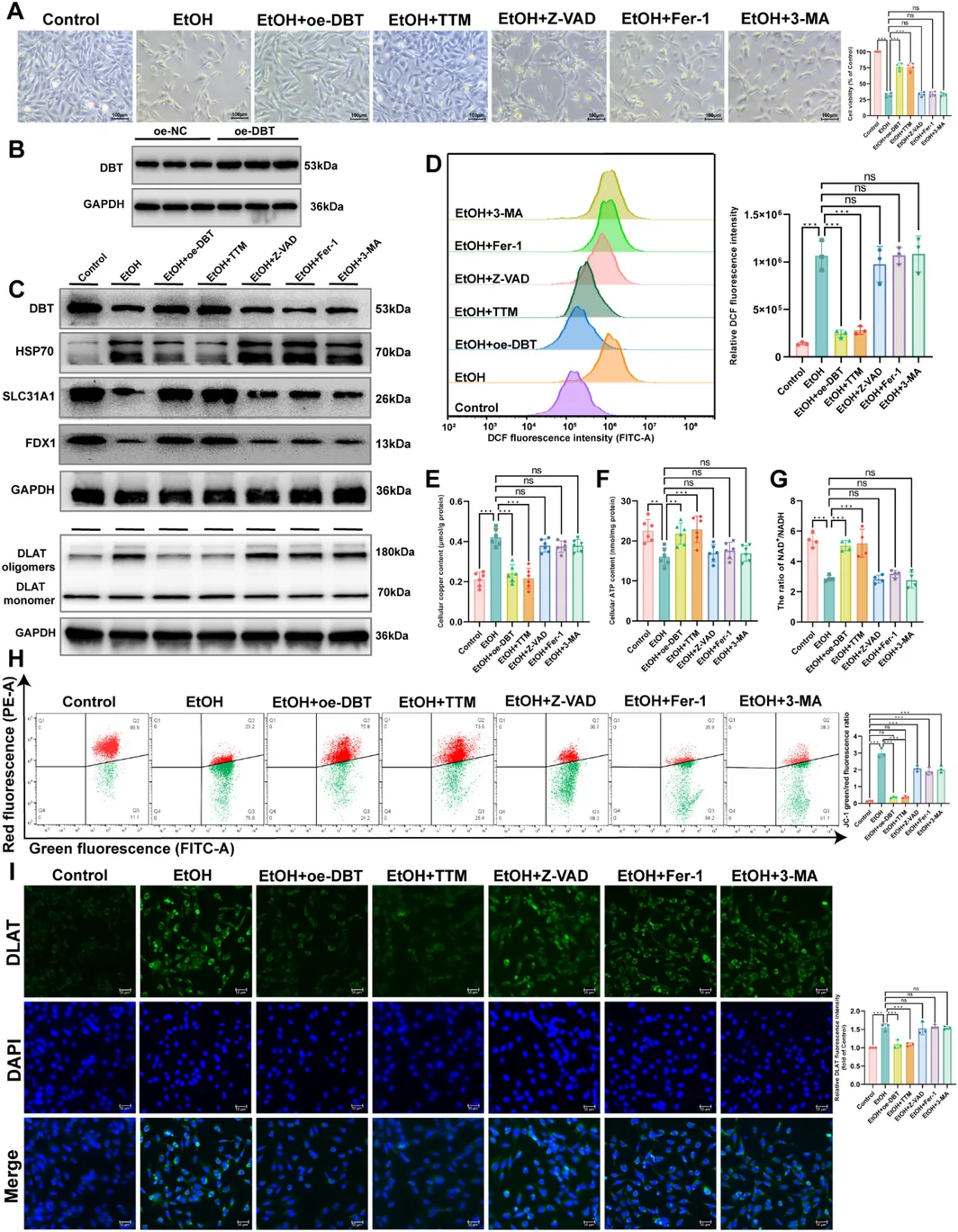
DBT overexpression attenuates EtOH‐induced cuproptosis‐related injury and mitochondrial dysfunction in AC16 cells. (A) Representative cell morphology and CCK‐8 analysis of cell viability in Control, EtOH, EtOH+oe‐DBT, EtOH+TTM, EtOH+Z‐VAD, EtOH+Fer‐1 and EtOH+3‐MA groups. Scale bar: 100 μm (*n* = 4). (B) Western blot validation of DBT overexpression efficiency in AC16 cells (*n* = 3). (C) Western blot analysis of DBT, HSP70, SLC31A1, FDX1, DLAT oligomers and DLAT monomer in AC16 cells (*n* = 3; corresponding WB quantification is shown in Figure S4D). (D) Flow cytometric analysis and quantification of intracellular ROS levels (*n* = 3). (E) Intracellular copper content in AC16 cells (*n* = 6). (F) Cellular ATP content (*n* = 6). (G) Cellular NAD^+^/NADH ratio (*n* = 4). (H) JC‐1 flow cytometric analysis and quantification of mitochondrial membrane potential, expressed as the green/red fluorescence ratio (*n* = 3). (I) Representative DLAT immunofluorescence staining and fluorescence intensity quantification in AC16 cells. Scale bar: 50 μm (*n* = 3) (Data are presented as mean ± SD. ns, not significant; *p < 0.05, **p < 0.01, ***p < 0.001).

DBT overexpression (Figure 7B) alleviated EtOH‐induced copper accumulation, cuproptosis‐related injury and mitochondrial dysfunction, while TTM showed similar protective effects. However, under the treatment conditions and sample sizes used in this study, Z‐VAD, Fer‐1, and 3‐MA did not produce statistically significant improvements in the above EtOH‐induced injuries.

## 4 Discussion

Alcoholic cardiomyopathy accounts for approximately one‐fifth of all cases of sudden cardiac death [47]. About 35% of non‐ischemic cardiomyopathies are attributable to excessive alcohol consumption [48]. Globally, alcohol overconsumption is responsible for 3 million deaths annually, accounting for 5% of total global mortality [49]. The pathogenic mechanisms by which ethanol induces ACM are multifactorial, including its direct cardiotoxic effects [50], as well as inflammation, oxidative stress, DNA damage and metabolic remodelling [51, 52]. Among these factors, mitochondrial dysfunction plays a central role in disease progression. This aligns well with cuproptosis, which involves mitochondrial protein aggregation and energetic collapse, suggesting that cuproptosis may serve as a link between ethanol exposure and myocardial injury. In this study, we provide evidence that cuproptosis may be involved in the pathogenesis of alcoholic cardiomyopathy.

We established a mouse ACM model using the EtOH‐Lieber‐Decarli liquid diet. RNA‐seq identified three cuproptosis‐related exploratory candidates: DBT, CP and PDK1. DBT encodes the E2 subunit of the branched‐chain α‐keto acid dehydrogenase complex and is considered a core regulator of cuproptosis [8, 53]. Although CP and PDK1 are also related to cuproptosis and mitochondrial metabolism [54, 55], respectively, qRT‐PCR and WB validation showed that only DBT exhibited significant differential expression in the ACM mouse model. Therefore, DBT was selected for further investigation.

In ACM mice, serum and cardiomyocyte copper levels increased with cuproptosis‐related molecular alterations. In AC16 cells, DBT knockdown produced similar alterations and mitochondrial dysfunction; TTM markedly rescued these changes, whereas MitoQ showed limited protection. Conversely, DBT overexpression attenuated EtOH‐induced cuproptosis‐related injury and mitochondrial dysfunction similarly to TTM. Z‐VAD, Fer‐1 and 3‐MA did not significantly improve EtOH‐induced injury under our conditions, but these findings do not exclude apoptosis, ferroptosis, autophagy, or other regulated cell‐death pathways. Together, these data support copper‐dependent cuproptosis‐related injury in AC16 cells and a regulatory role for DBT at the cellular level, while the in vivo findings remain associative.

DBT encodes the E2 subunit of the branched‐chain α‐keto acid dehydrogenase complex (BCKDH), which mediates BCAA catabolism. Impaired BCAA catabolism can disrupt glucose oxidation and mitochondrial pyruvate utilization, contributing to cardiac metabolic dysfunction [56]. BCAA catabolites enter the TCA cycle, while BCAAs also regulate cardiac metabolic signalling [45]. Because cuproptosis involves mitochondrial lipoylated proteins [8], altered BCKDH‐dependent metabolism may influence cuproptosis susceptibility. However, BCKDH activity, BCAA metabolic flux, mitochondrial respiration and TCA‐cycle activity were not assessed. This proposed metabolic link therefore remains hypothetical; our data also do not establish that copper directly induces DBT degradation or that DBT buffers intracellular copper.

Consistent with this potential metabolic link, reduced DBT expression has been associated with dilated cardiomyopathy [57] and heart failure [58]. Our findings further suggest that DBT may link mitochondrial metabolic dysfunction to cuproptosis‐related cardiomyocyte injury and contribute to ACM pathogenesis. However, this mechanistic relationship remains to be fully established, and the direct causal role of DBT in vivo requires further validation.

Despite the evidence from in vivo and in vitro models supporting the role of DBT in ethanol‐induced cuproptosis‐related cardiomyocyte injury, several limitations should be acknowledged. (1) Cardiomyocyte‐specific DBT overexpression or knockdown/knockout models were not established. Thus, the in vivo data support associations among DBT downregulation, copper accumulation, cuproptosis‐related alterations and ACM, but do not establish a causal role of cuproptosis in cardiac dysfunction or ACM progression, or of DBT acting through cuproptosis in vivo. Further validation using cardiomyocyte‐specific DBT manipulation and in vivo cuproptosis‐targeted intervention and rescue is required. (2) ROS, ATP, NAD^+^/NADH and mitochondrial membrane potential were assessed to characterize mitochondrial dysfunction, but metabolic flux, mitochondrial respiration, TCA‐cycle activity and BCKDH activity were not directly measured. Thus, whether DBT alters cuproptosis susceptibility through BCKDH and BCAA/TCA metabolism remains unclear. (3) Although this study combined a murine ACM model with human AC16 cells, interspecies differences in cardiac mitochondrial metabolism, redox responses and stress adaptation may affect the DBT‐mitochondrial dysfunction‐cuproptosis relationship and its translation to human ACM [59]. AC16 cells also do not fully recapitulate primary human cardiomyocytes or the complex pathology of human ACM. Further validation in more physiologically relevant human models and clinical samples is therefore needed. (4) Only male mice were used in this study to reduce biological variability caused by the estrous cycle and hormonal fluctuations in female mice. However, sex differences may affect alcohol metabolism, inflammatory responses, mitochondrial function and cell death processes. Therefore, future studies should include female animals or animals at different ages to further validate the generalizability of our findings. (5) Although Z‐VAD, Fer‐1 and 3‐MA showed no significant protection, these negative results cannot exclude apoptosis, ferroptosis, or autophagy, or define their relative contributions to ACM. (6) No prospective power calculation was performed, and some mechanistic experiments included only three independent biological replicates, potentially limiting statistical power. Thus, negative or borderline findings should be interpreted cautiously and cannot exclude the corresponding mechanisms.

## 5 Conclusions

This study provides new evidence supporting the potential involvement of cuproptosis in ACM‐associated myocardial injury. Through RNA‐seq, bioinformatics analysis and functional experiments, DBT was identified as an exploratory cuproptosis‐related candidate in ACM. In AC16 cells, DBT downregulation promoted cuproptosis‐related cardiomyocyte injury and mitochondrial dysfunction, whereas DBT overexpression alleviated EtOH‐induced cuproptosis‐related injury and mitochondrial dysfunction. TTM rescue further supported the copper dependence of this process. These findings suggest that DBT may serve as a potential therapeutic target for mitigating alcohol‐induced myocardial injury by regulating cuproptosis‐related processes at the cellular level. However, whether DBT directly regulates ACM progression through cuproptosis in vivo remains to be determined.

## Author Contributions

**Zhihao Pan:** supervision, project administration, funding acquisition, conceptualization, writing – review and editing. **Liuming Gao:** methodology, validation, writing – review and editing. **Yi Ling:** resources, data curation, writing – review and editing. **Xianfeng Cen:** supervision, project administration, funding acquisition, conceptualization, writing – review and editing.

## Funding

Doctoral Start‐up Research Fund of the Affiliated Lihuili Hospital of Ningbo University (No. 2023BSKY‐CXF).

## Ethics Statement

All animal experiments were approved by the Animal Ethics Committee of the Medical Research Institute, Wuhan University (Approval No. MRI2021‐LAC18), and were performed in accordance with the institutional guidelines and the National Institutes of Health Guide for the Care and Use of Laboratory Animals.

## Consent

The authors have nothing to report.

## Conflicts of Interest

The authors declare no conflicts of interest.

## Supporting information


**Figure S1:** Density plot and violin plot of FPKM.

**Figure S2:** Independent RNA‐seq validation of DBT expression in ACM.

**Figure S3:** Representative images of isolated mouse cardiomyocytes.

**Figure S4:** Supplementary quantification of NAD^+^, NADH and Western blot analyses.

**Table S1:** List of cuproptosis‐related genes.

**Table S2:** List of primers for qRT‐PCR.

**Table S3:** Information on antibodies used in this study.

**Table S4:** Exploratory cuproptosis‐related candidate genes identified in the discovery and validation RNA‐seq cohorts.

## Data Availability

All datasets generated or analysed during this study are included in this published article and its additional information files, which are publicly accessible. All the raw codes are available from the corresponding author.
